# *Mentha rotundifolia* (L.) Huds. and *Salvia officinalis* L. hydrosols mitigate aging related comorbidities in rats

**DOI:** 10.3389/fnagi.2024.1365086

**Published:** 2024-02-23

**Authors:** Khadija Boualam, Hind Ibork, Zakaria Lahboub, Mansour Sobeh, Khalid Taghzouti

**Affiliations:** ^1^AgroBioSciences Program, College of Agriculture and Environmental Sciences, University Mohammed VI Polytechnic, Ben-Guerir, Morocco; ^2^Physiology and Physiopathology Team, Genomics of Human Pathologies Research Center, Faculty of Sciences, Mohammed V University in Rabat, Rabat, Morocco; ^3^Plant Chemistry and Organic and Bioorganic Synthesis Team, Chemistry Department, Faculty of Sciences, Mohammed V University in Rabat, Rabat, Morocco

**Keywords:** *Mentha rotundifolia*, *Salvia officinalis*, aging, hydrosols, healthy aging

## Abstract

**Introduction:**

Aging is often linked to oxidative stress, where the body experiences increased damage from free radicals. Plants are rich sources of antioxidants, playing a role in slowing down aging and supporting the proper functioning and longevity of cells. Our study focuses on exploring the impact of *Mentha rotundifolia* (MR) and *Salvia officinalis* (SO) hydrosols on aging-related comorbidities.

**Methods:**

The chemical composition of MR and SO hydrosols was analyzed by gas chromatography coupled to mass spectrometry. 2,2-Diphenyl 1-picrylhydrazyl and 2,20-azino-bis 3-ethylbenzothiazoline-6-sulfonic acid radicals scavenging assays were used to assess their *in vitro* antioxidant activity, and heat induced albumin denaturation test was used to evaluate their anti-inflammatory activity. Subsequently, we administered 5% of each plant hydrosol in the drinking water of 18-month-old rats for six months. We then conducted behavioral tests, including open field, dark/light box, rotarod, and Y-maze assessments, and measured biochemical parameters in plasma, liver and brain tissues.

**Results and discussion:**

At two years old, animals treated with MR and SO hydrosols displayed fewer physical and behavioral impairments, along with well-preserved redox homeostasis in comparison with animals in the control group. These results highlighted the significance of MR and SO hydrosols in addressing various aspects of age-related comorbidities. The study suggests that these plant-derived hydrosols may have potential applications in promoting healthy aging and mitigating associated health challenges.

## Graphical abstract

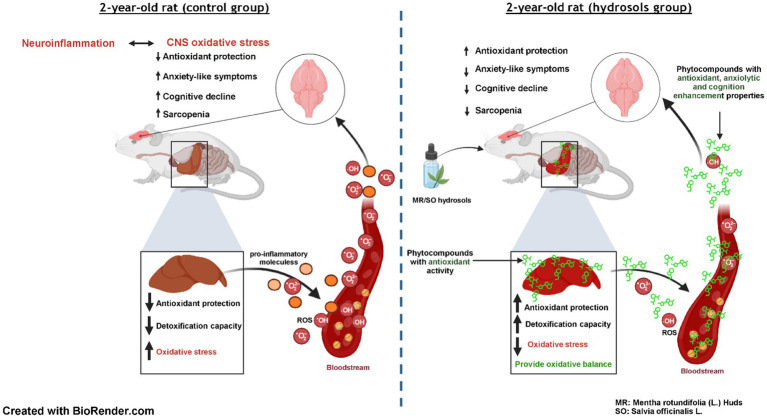

## 1 Introduction

Aging is a complex physiological process that affects all living organisms, including humans and animals. It is characterized by a progressive decline in physiological function, resulting in an increased susceptibility to age-related diseases and ultimately, death (Khan et al., 2017). Aging is driven by a combination of genetic, environmental, and lifestyle factors, including oxidative stress, inflammation, telomere shortening, mitochondrial dysfunction, and cellular senescence (Song et al., 2020).

The oxidative stress theory of aging suggests that one of the key drivers of the aging process is the accumulation of reactive oxygen species (ROS) in cells (Lin and Flint Beal, 2003). ROS are generated during normal cellular metabolism and can cause damage to cellular structures, including lipids, proteins, and DNA (Checa and Aran, 2020). Over time, this damage accumulates and contributes to the development of aging-related comorbidities such as cardiovascular disease, cancer, and neurodegenerative disorders (Ziada et al., 2020).

While aging cannot be prevented, it is possible to slow down the process and delay the onset of aging-related diseases (Poeggeler, 2005). In this context, bioactive compounds from plants with antioxidant and anti-inflammatory properties might represent a promising approach (Barrajón-Catalán et al., 2014; Spagnuolo et al., 2018; Proshkina et al., 2020). Furthermore, phyto-molecules exhibit extensive structural diversity and demonstrated the capability to interact with and bind to multiple receptors, thereby engaging in multitarget interactions (Panossian and Efferth, 2022). This attribute confers polyvalent pharmacological actions and pleiotropic therapeutic activities.

Plant hydrosols have been extensively investigated for their antioxidant and antimicrobial properties in food and cosmetic industries (Catty, 2001; Wajs-Bonikowska et al., 2015). Nevertheless, their potential pharmacological effects have been acknowledged within the realm of traditional medicine. Functioning as a mild form of aromatherapy, these hydrosols feature minimal concentrations of essential oil molecules (less than 1 g/L), rendering them both secure and user-friendly (Acimovic et al., 2020). Several hydrosols are used as functional drinks for the mitigation of infections, hormonal imbalances, depressive states, sleep disturbances, digestive ailments, and neural illnesses (Yaghoob et al., 2004; Baydar et al., 2013; Kunicka-Styczyńska et al., 2015; Hamedi et al., 2017a,b).

In the light of these data, we aim to evaluate the anti-aging effects of hydrosols derived from two Lamiaceae plants: *Mentha rotundifolia* (L.) Huds (MR) and *Salvia officinalis* L. (SO). The selection of these plant species is substantiated by their well-documented antioxidant, anti-inflammatory, neuroprotective, and analgesic effects (Hasanein et al., 2016; Boualam et al., 2023). We here assessed the *in-vitro* antioxidant and antiinflammatory properties of MR and SO hydrosols. Subsequently, we administered 5% of each plant hydrosol in the drinking water provided to 18-month-old Wistar rats over a period of six months. Upon reaching an age of 24-month-old, we conducted a thorough examination of physical, behavioral, and biochemical changes in the aged rat cohort. Finally, the phytoconstituents present in the plant hydrosols were characterized using gas chromatography coupled with mass spectrometry (GC–MS) technique.

## 2 Materials and methods

### 2.1 Extraction process

The aerial parts from SO and MR were harvested during their blooming periods (June and September respectively), from Mkam Tolba (33°55′41.9” N, 6°16′34.2” W) in Khemisset, Morocco. The plant samples were dried in shade at room temperature for two weeks. 500 g of each plant powder was distilled separately in 2 L using a Clevenger apparatus for 3 h. At the end of the hydrodistillation process, the essential oils have been removed and 500 mL of each plant’s hydrosols was collected.

### 2.2 Chemical composition

The composition of MR and SO hydrosols was analyzed using Nexis 2030 gas chromatography system coupled to a TQ8040 NX mass spectrometer (GCMS-TQ8040, SHIMADZU, JAPAN). The separation of the compounds was performed using a Restek RTX-5MS column (30 0.25 mm, film thickness 0.25 μm). The GC temperature was programmed at 50°C for 2 min and increased to 300°C with a rate of 5.5 C/min and stabilized for 3 min at 300°C. Helium was used as the carrier gas with a flow rate of 1.5 mL/min. The sample (1 μL) was injected in split mode (HTA 2800 T injector, HT 250 C). The mass scan was *m/z*: 50–500. The EO compounds are identified by their column retention indices (RI) determined with reference to the C_5_-C_24_ (n-alkane) homolog series, and by comparison of their mass spectra with literature reports using the NIST 2017 and Wiley version libraries (Wiley and NIST, 2017).

### 2.3 Blood brain barrier and gastro-intestinal absorption of characterized compounds

Swiss ADME^1^ tool was used to verify the gastro-intestinal (GI) and the blood brain barrier (BBB) absorptions of the characterized compounds (Daina et al., 2017).

### 2.4 Antioxidant activity

#### 2.4.1 DPPH radical scavenging activity

2,2-diphenyl 1-picrylhydrazyl (DPPH) solution was prepared in methanol at the concentration of 79 μg/mL. Then, MR and SO hydrosols solutions were prepared at concentrations of 50–1,000 μL/mL and were mixed with 0.5 mL of DPPH. The reaction mixture was then incubated for 30 min at room temperature in the dark, followed by measurement of the absorbance at 517 nm (Boualam et al., 2023). The scavenging activity was estimated as a percentage using the following formula:


$$\mathrm{Free radical scavenging}\;\left(\%\right)=\frac{O.D\;\mathrm{control}-O.D\;\mathrm{test}}{O.D\;\mathrm{control}}\times 100$$

where O.D control and O.D test represent the absorbance values of the control and test samples, respectively.

#### 2.4.2 ABTS+ scavenging activity

ABTS assay involves stabilizing the blue-green colored cationic radical 2,20-azino-bis 3-ethylbenzothiazoline-6-sulfonic acid (ABTS+) by an antioxidant, which results in the transformation of the radical to a colorless form. A mixture was prepared by adding 1.9 mL of ABTS+ solution to 600 μL of MR and SO hydrosols solution at various concentrations ranging from 50 to 1,000 μL/mL. It was then incubated at room temperature for 7 min and the absorbance was measured at 734 nm (Boualam et al., 2023). To obtain a blank reading, methanol was used instead of the hydrosols solution. The scavenging activity was estimated as a percentage using the following formula:


$$\mathrm{Free radical scavenging}\;\left(\%\right)=\frac{O.D\;\mathrm{control}-O.D\;\mathrm{test}}{O.D\;\mathrm{control}}\times 100$$

where O.D control and O.D test represent the absorbance values of the control and test samples, respectively.

### 2.5 Animals and study design

Wistar rats (18-month-old) weighing 400–450 g were selected for the following experiment. They were raised at the central animal care facilities of the Faculty of Sciences, Mohammed V University of Rabat, Morocco and were housed in polyethylene cages in a room with controlled temperature (22 ± 1°C), under a 12 h light–dark cycle. Animals had free access to food and water and were fed on a normal chow diet (Cicalim, Casa Blanca). After two weeks acclimatization, the animals were randomly divided into three groups of six rats each. Due to the chronicity of the treatment period, the treated groups received hydrosols in drinking water to avoid the complications related to long-term oral gavage, namely stress, aspiration pneumonia, unintentional tracheal administration, esophageal trauma, and gastric rupture (Kinder et al., 2014; Dhawan et al., 2018).

- Group 1: Control group, received tap water
- Group 2: received 5% of MR hydrosol in drinking water for six months
- Group 3: received 5% of SO hydrosol in drinking water for six months

**Figure 1:**
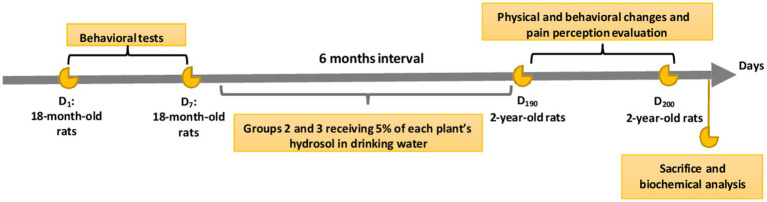
Overview of the study design.

Prior to the administration of the hydrosols, the behavioral tests (open field, dark/light box, rotarod, and Y-maze) were performed for all groups, then on day 8, the hydrosols were added to the drinking water of groups 2 and 3 (Figure 1). After a six-month treatment period, we assessed various signs of aging, including physical, cognitive, and biochemical indicators.

### 2.6 Physical changes analysis

At the age of 2 years, the physical changes between the animals treated with the hydrosols and the control rats were analyzed. An observer who was not informed of the study protocol was asked to identify differences. Rats, like other animals, exhibit several signs of aging as they grow older. Some common physical signs of aging in rats include changes in posture, fur, and muscle mass (Phillips et al., 2010). As rats age, they may develop a hunched or stooped posture due to the loss of muscle mass and strength (Baek et al., 2020). This may also impact eyelid muscles leading to ptosis. Their fur may become less shiny and coarse following a lack of grooming routine related to aging (Scimonelli et al., 1999). Additionally, they may experience hair loss in some areas.

### 2.7 Behavioral tests

#### 2.7.1 Open field

The open field test (OF) is a widely utilized method for measuring exploratory behavior and general locomotor activity in mice and rats, as well as assessing anxiety-related behaviors by monitoring defecation and visited areas (Gould et al., 2009; Seibenhener and Wooten, 2015). The OF apparatus employed in this study consists of a black square with an 80 cm^2^ surface area and a height of 45 cm. The ground of the field is divided into 25 squares of 15 cm × 15 cm each by white strips. The test is conducted in a dimly lit room, and each animal is placed at the center of the field. The field is cleaned after each test, and each animal is allowed to explore the field for 10 min. Locomotor activity is assessed by measuring the distance the animal covers (i.e., the number of tiles crossed) and the number of sit-ups performed. Anxiety-related behavior is evaluated by calculating the number of entries into the central zone of the OF and the duration of time spent in this area.

#### 2.7.2 Rotarod test

The assessment of motor coordination in rodents is commonly achieved using the rotarod test (Shiotsuki et al., 2010). The test apparatus consists of a plastic rotating cylinder, measuring 7 cm in diameter and 28 cm in length that is fixed around a horizontal axis elevated by 30 cm and divided into four compartments with a width of 9 cm each (Panlab, Harvard Apparatus). The test is conducted in an isolated room with normal lighting and is composed of two phases: a pre-test phase, during which the animals are habituated to the apparatus, and a test phase, during which rats are placed on the rotating cylinder at a fixed speed of 4 rotations per min (rpm). Once the animal is stabilized, the speed of rotation is progressively increased until reaching a maximum speed of 40 rpm, which is then maintained until the end of the test. The duration of the test is limited to 180 s to prevent fatigue or stress-related effects (Shiotsuki et al., 2010).

#### 2.7.3 Dark/light box test

The dark/light box test is a commonly used protocol to measure anxiety-like behavior in rodents, involving a two-chambered apparatus consisting of a small dark compartment connected to a larger well-lit compartment by an opening, which the animal can freely explore (Crawley, 1985). In this test, animals typically show a natural aversion to the brightly lit area and prefer to remain in the dark chamber. The test is conducted in a dimly lit room, and the animal is initially placed in the dark compartment and allowed to explore both compartments for 10 min. The amount of time spent in the light compartment and the number of crossings between the two compartments are recorded as measures of anxiety-like behavior.

#### 2.7.4 Y-maze test

The Y-maze test is a reliable method for evaluating both spatial working and reference memories in rodents. The Y-maze device consists of a black Y-shaped apparatus with three arms arranged at 120° angles from each other. To assess spatial working memory, spontaneous alternation is calculated by recording the number of arm entries and consecutive entries into all three arms. The percentage of alternation behavior is determined using the formula (Kraeuter et al., 2019):


$$\%\mathrm{Alternation}=\left(\begin{array}{l} \mathrm{Number of Alternations}/\\ \left[\mathrm{Total number of}\;\mathrm{arm}\;\mathrm{entries}-2\right] \end{array}\right)\times 100.$$

To assess reference spatial memory, the test is organized into two phases: a training session, during which one arm of the Y-maze is closed off and designated as the novel arm, and a test session, which takes place after a 1-h time interval. During the test session, the animal is placed back in the Y-maze device and is expected to remember the location of the novel arm and visit it more frequently than the other arms. The number of entries into the novel arm is compared to the entries into the other arms to determine the degree of spatial memory (Kraeuter et al., 2019).

### 2.8 Tail immersion test for hyperalgesia

According to Taïwe and Kuete (2017), hyperalgesia is characterized by an increased sensitivity to pain induced by noxious or ordinarily non-noxious stimulation of peripheral tissues. The tail immersion test, first described by Janssen et al. (1963), is a commonly used method for assessing hyperalgesia. In this test, animals are restrained, and the tip of their tails is immersed in a water bath at a temperature of 50 ± 0.5°C. The tail withdrawal reflex, which is mediated by spinal nociceptive reflexes (Bannon and Malmberg, 2007), is elicited as a response to the heat stimulus, and the latency of this reflex is measured. To avoid potential skin damage, each immersion is terminated after 15 s.

### 2.9 Acetone test for cold allodynia

The Acetone test was used to assess cold allodynia (Deuis et al., 2017). A drop of acetone was applied to the plantar surface of each hind paw using a pipette. Rats were then placed in individual transparent chambers and the time spent licking, biting or lifting the paw was recorded for 2 min. The same procedure was repeated 3 times at 5 min intervals and the results were averaged. Control tests were performed using saline instead of acetone. An increase in the time spent licking, biting or lifting the paw compared to control tests may indicate cold allodynia.

### 2.10 Biochemical analysis

All animals were sacrificed by decapitation 24 h after the completion of the last test and following an overnight fast. Trunk blood samples were collected in heparinized sample bottles and centrifuged at 2000 rpm for 10 min to obtain plasma which was used for biochemical analysis. The plasma levels of alanine transaminase (ALT), aspartate transaminase (AST), bilirubin, and gamma-glutamyl transferase (GGT) were measured using standard methods. Tissue samples were collected from experimental animals and kept chilled on ice throughout the procedure. Brain and liver tissues were homogenized in 50 mM phosphate buffer, pH 7.4 and centrifuged at 4000 rpm for 15 min at 4°C to obtain the soluble fraction (Mahmoud et al., 2023). Supernatants were collected and protein content was determined by adding alkaline copper tartrate solution and Folin–Ciocalteu reagent. Then, absorbance was measured at 750 nm following the method of (Lowry et al., 1951). Bovine Serum Albumin (BSA) was used as a standard. Superoxide dismutase (SOD), Glutathione peroxidase (GPx), and Glutathione (GSH) concentrations were measured by the corresponding biochemical kits (ab285309, ab281168, and ab239727, Abcam, US) according to the manufacturers’ instructions.

### 2.11 Statistical analysis

Data were analyzed using GraphPad Prism 8. Normality was assessed by the Shapiro–Wilk test. IC_50_ values were presented as means ± SD (*n* = 3) and were compared using *t*-test. Behavioral tests results were analyzed using two-ways ANOVA considering ‘treatment’ and ‘age’ as variation factors. Biochemical analyses were analyzed using one-way ANOVA. Bonferroni’s multiple comparisons test was used whenever there was significance. Results are expressed as means ± SD (*n* = 6). The differences were regarded as statistically significant at *p* ≤ 0.05.

## 3 Results

### 3.1 Chemical composition and pharmacokinetics parameters

**Figure 2:**
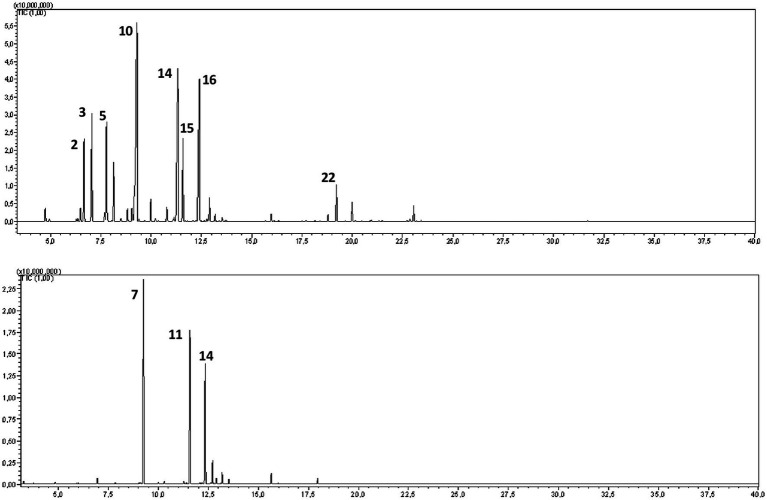
GC–MS chromatograms of the hydrosols of *Salvia officinalis* (SO, top) and *Mentha rotundifolia* (MR, bottom).

GC–MS analysis of the hydrosols revealed the presence of numerous aromatic compounds, some present in substantial concentrations (Figure 2; Table 1). SO contained notable amounts of α-pinene, camphene, 1,8 cineole, and camphor (Table 1), while MR hydrosols displayed richness in camphor, 1,8 cineole, and (E)-thujone (Table 1). Except for α-pinene and camphene, the identified molecules are characterized by favorable GI and BBB absorption properties (Table 1).

**Table 1:** Annotated phytocompounds from hydrosols of *Salvia officinalis* (SO) and *Mentha rotundifolia* (MR) with their pharmacokinetics parameters.

N°	Compound	Ri calculated	Ri reported	Relative abundance	GI absorption	BBB absorption
*Salvia officinalis* (SO)						
1	α-Thujene	914	925	0.84	Low	Yes
2	α-Pinene	923	934	5.17	Low	Yes
3	Camphene	932	947	7.08	Low	Yes
4	Sabinene	958	967	0.58	Low	Yes
5	β-Pinene	965	973	6.75	Low	Yes
6	β-Myrcene	977	989	1.4	Low	Yes
7	α-Phellandrene	996	1,004	0.2	Low	Yes
8	(+)-4-Carene	1,000	1,007	0.81	Low	Yes
9	m-Cymene	1,013	1,022	1.25	Low	Yes
10	1,8-Cineole	1,024	1,026	24.73	Yes	Yes
11	ɣ-Terpinene	1,048	1,059	1.22	Low	Yes
12	Sabinene hydrate	1,056	1,060	0.19	Yes	Yes
13	Isoterpinolene	1,080	1,079	0.86	Low	Yes
14	Thujone (Z)	1,103	1,102	17.08	Yes	Yes
15	Thujone (E)	1,113	1,114	9.59	Yes	Yes
16	D-camphor	1,146	1,143	14.16	Yes	Yes
17	(+)-Borneol	1,158	1,153	1.48	Yes	Yes
18	(−)-Terpinen-4-ol	1,170	1,164	0.44	Yes	Yes
19	L-α-Terpineol	1,182	1,175	0.21	Yes	Yes
20	Bornyl acetate	1,278	1,270	0.45	Yes	Yes
21	Methyl eugenol	1,388	1,376	0.40	Yes	Yes
22	Caryophyllene	1,416	1,407	2.2	Low	No
23	α-humulene	1,451	1,449	1.18	Low	No
24	Caryophyllene oxide	1,557	1,570	0.14	Yes	Yes
25	Viridiflorol	1,596	1,579	0.96	Yes	Yes
*Mentha rotundifolia* (MR)						
1	Hexanal	792	801	0.16	Yes	Yes
2	3-Hexen-1-ol, (Z)	836	842	0.42	Yes	Yes
3	Thuja-2,4(10)-diene	952	953	0.92	low	yes
4	1-Octen-3-ol	961	974	0,24	Yes	Yes
5	3,6-Heptadien-2-ol	987	999	0.09	Yes	Yes
6	m-Cymene	1,010	1,020	0.26	Low	Yes
7	1,8 cineole	1,019	1,026	34.45	Yes	Yes
8	Beneneacetaldehyde	1,033	1,042	0.1	Yes	Yes
9	Thujone (Z)-	1,101	1,102	0.41	Yes	Yes
10	1-Octen-3-yl-acetate	1,105	1,113	0.25	Yes	Yes
11	Thujone (E)-	1,113	1,114	26.97	Yes	Yes
12	Limonene oxide (Z)	1,132	1,137	0.27	Yes	Yes
13	Sabinol (Z)	1,138	1,143	0.25	Yes	Yes
14	Camphor	1,144	1,146	20.57	Yes	Yes
15	Pinocarvone	1,156	1,165	0.17	Yes	Yes
16	*p*-Mentha-1,5-dien-8-ol	1,159	1,166	3.67	Low	Yes
17	Isoborneol	1,166	1,173	1.25	Yes	Yes
18	Terpinen-4-ol	1,178	1,177	1.94	Yes	Yes
19	L-α-terpineol	1,192	1,192	0.9	Yes	Yes
20	Myrtenol	1,199	1,194	0.07	Yes	Yes
21	Verbenone	1,204	1,206	0.15	Yes	Yes
22	L-bornyl acetate	1,290	1,283	0.18	Yes	Yes
23	Piperitenone oxide	1,369	1,366	0.88	Yes	Yes

### 3.2 Antioxidant activity

MR and SO hydrosols exhibited an interesting scavenging ability against DPPH and ABTS radicals (IC_50_ ≤ 100 μg/mL), Table 2. The obtained results may be attributed to the presence of terpenes with antioxidant behavior such as 1,8 cineole, camphor, and caryophyllene.

**Table 2:** IC_50_ values of the hydrosols of *Salvia officinalis* (SO) and *Mentha rotundifolia* (MR) in comparison with ascorbic acid in DPPH and Trolox in ABTS assays.

Hydrosols	DPPH	ABTS+
	IC_50_ (μg/mL)	
MR hydrosol	95.54 ± 1.91^ab^	101.097 ± 2.04^ab^
SO hydrosol	87.194 ± 2.72^a^	82.315 ± 0.22^a^
Ascorbic acid	1.93 ± 1.47	-
Trolox	-	5.21 ± 0.64

Values are presented as mean ± SD of triplicate readings (*n* = 3). The results are considered significantly different for *p* ≤ 0.05. ^a^ in comparison with the standard, ^b^ in comparison with SO hydrosol.

### 3.3 Protein denaturation inhibition assay

**Figure 3:**
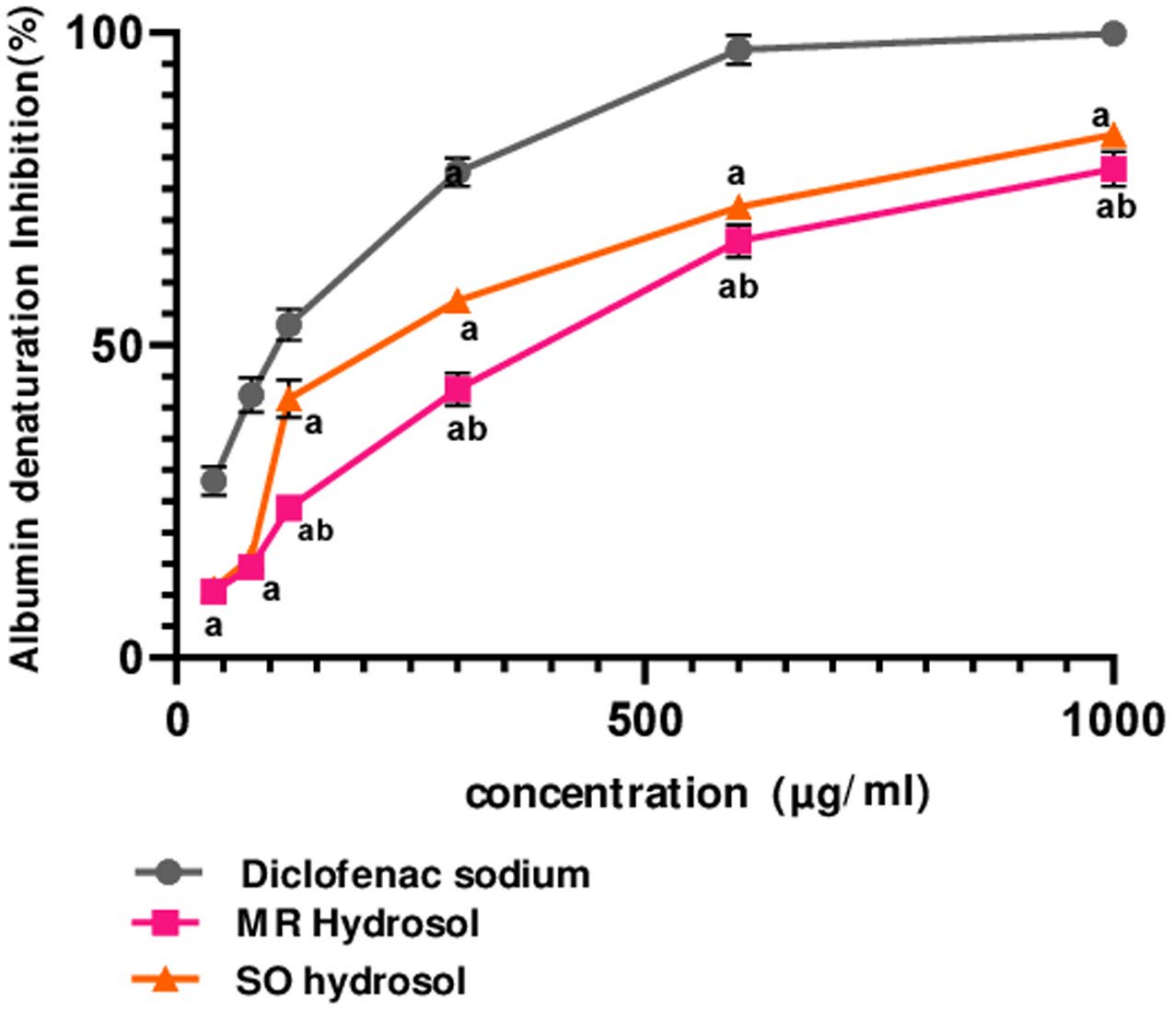
Inhibitory effects of hydrosols *Salvia officinalis* (SO, IC_50_ = 230.6 ± 0.44 μg/mL) and *Mentha rotundifolia* (MR, IC_50_ = 346 ± 1.73 μg/mL) on albumin denaturation in comparison with diclofenac (IC_50_ = 98.63 ± 2.18 μg/mL). Data were analyzed using two-ways ANOVA followed by Bonferroni post-hoc test and were considered as statistically different for *p* ≤ 0.05. ^a^ in comparison with the standard, ^b^ in comparison with SO hydrosol. Data are presented as (mean ± SD) for *n* = 3.

The *in-vitro* anti-inflammatory activity of MR and SO hydrosols was evaluated using a thermally induced protein denaturation protocol. Both hydrosols, along with the standard diclofenac, exhibited a dose-dependent anti-inflammatory effect (Figure 3). Remarkably, starting from 120 μg/mL, SO hydrosol demonstrated higher activity compared to MR hydrosol (*p* ≤ 0.05), a finding corroborated by its significantly lower IC_50_ value (*p* ≤ 0.05) (Figure 3).

### 3.4 Physical changes analysis

A qualitative physical analysis was performed. Apparent aging signs were evaluated including posture, eyelids muscle, and fur changes, Table 3. All groups presented some or all aging signs as it’s an inevitable process. However, there were substantial differences between the control group and hydrosols treated groups. Treatment with hydrosols prevented aging-related muscle loss, enabling treated animals to maintain firmer posture and more open eyelids (Table 3).

**Table 3:** Effects of the hydrosols of *Salvia officinalis* (SO) and *Mentha rotundifolia* (MR) on aging signs identified in 24-month-old rats.

Treatments	Fur aspect			Hunched Posture	Ptosis
	Dull	Coarse	Hair loss		
Control group	+++	+++	+++	++	++
MR hydrosol	+	++	++	+	−
SO hydrosol	−	+	++	−	−

+++: Present in all individuals of the group, ++: Present in the majority/half of individuals in the group, +: Present in the minority of individuals in the group, −: absent.

### 3.5 Behavioral changes

#### 3.5.1 Hydrosols ameliorate locomotion and motor coordination impairments in aging rats

**Figure 4:**
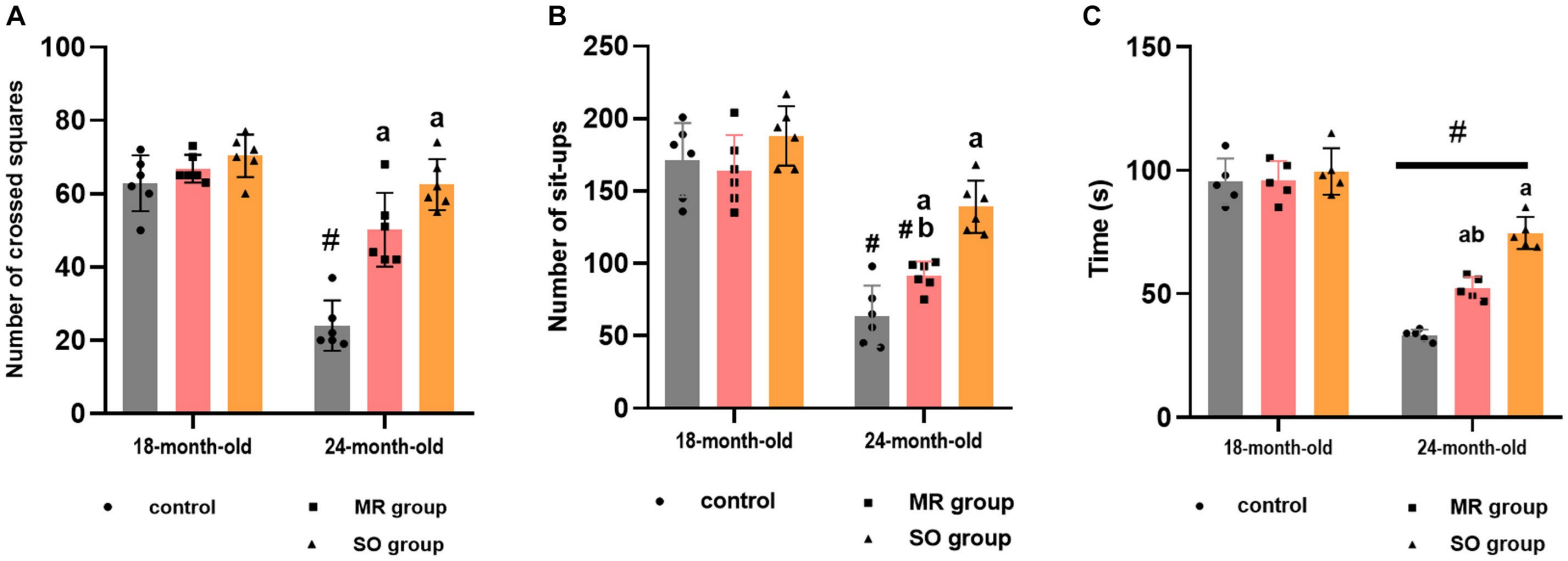
Effects of the hydrosols of *Salvia officinalis* (SO) and *Mentha rotundifolia* (MR) on the number of crossed squares **(A)**, the number of set-ups of aged rats in the open field **(B)**; and their time passed on the carrousel of rotarod apparatus **(C)**. Results are presented as means ± SD (*n* = 6). ^#^*p* ≤ 0.05 compared to the same young rat groups (control, MR, and SO groups at 18-month-old). ^a^*p* ≤ 0.05 compared to aged control rats (24- month-old). ^b^*p* ≤ 0.05 compared to SO-aged treated rats.

The impact of treatment with SO and MR hydrosols on aging-related locomotion and motor coordination impairments was evaluated through the open field task and rotarod test. Performance metrics, such as the number of crossed squares and sit-ups in the open field, as well as the duration spent on the rotarod, were quantified (Figure 4). In the open field test, a significant reduction in the number of crossings was observed in aged rats compared to their younger counterparts (*p* < 0.0001). On the other hand, the administration of both SO and MR hydrosols resulted in a substantial improvement in the number of crossings in aged rats (*p* < 0.0001 for both groups) (Figure 4A). Similarly, the number of sit-ups significantly decreased in the aged group (*p* < 0.0001) for both control and MR groups compared to younger rats. Notably, in the SO hydrosol-treated group, no significant difference was observed (*p* = 0.1636) compared to the control group (Figure 4B), indicating an ameliorative effect. Furthermore, the time spent on the rotarod by young rats significantly exceeded that of aged rats (*p* < 0.0001). Aging rats treated with SO hydrosol exhibited a significant increase in performance on the rotarod compared to the control aged group (*p* < 0.0001) and the MR aged group (*p* = 0.0007) (Figure 4C).

#### 3.5.2 Hydrosols alleviate anxiety-like symptoms in aging rats

**Figure 5:**
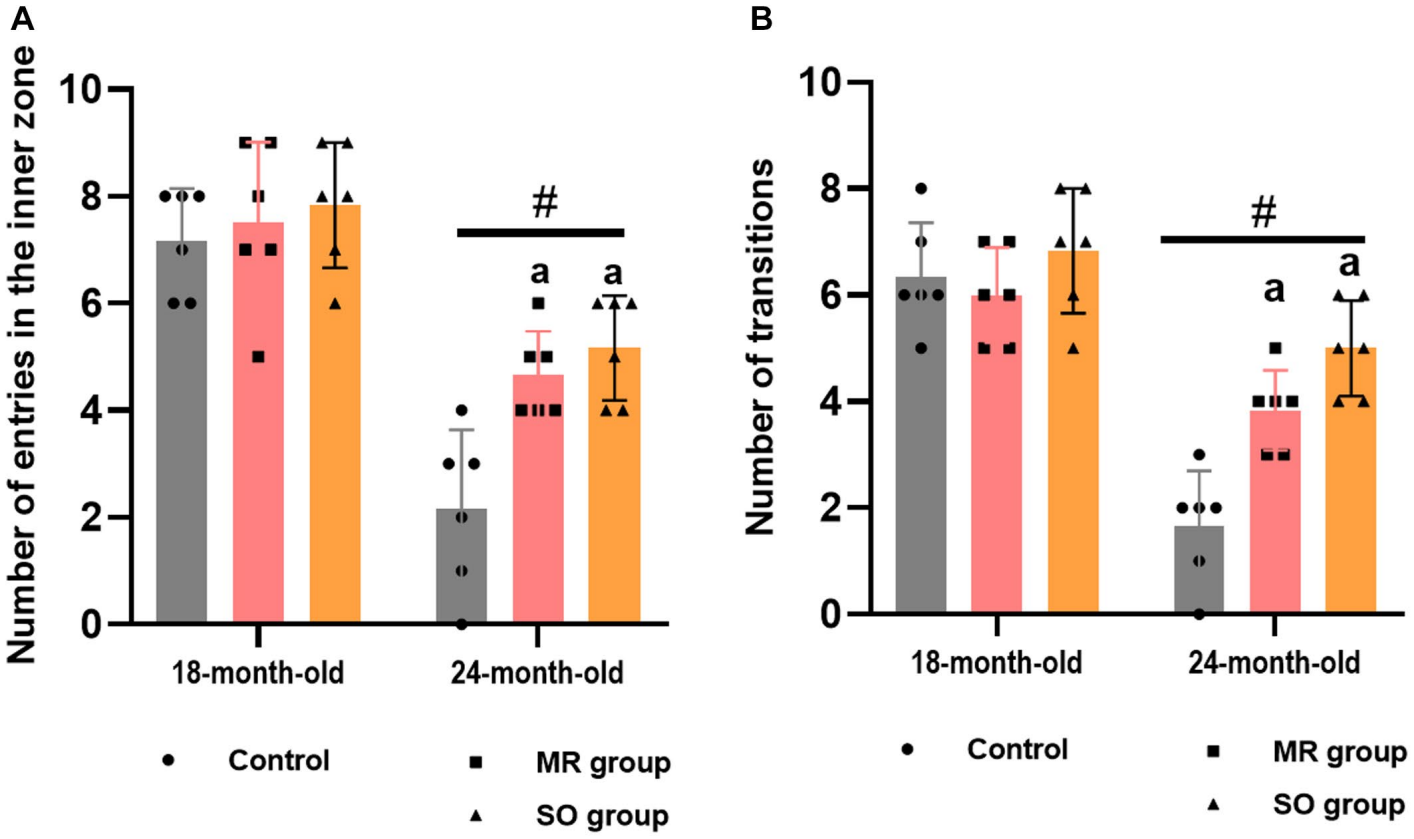
Effects of the hydrosols of *Salvia officinalis* (SO) and *Mentha rotundifolia* (MR) on the number of rats entries in the inner zone in the open field **(A)**, and the number of transitions in the light/dark box task **(B)**. Results are presented as means ± SD (*n* = 6). ^#^*p* ≤ 0.05 compared to the young rat groups (control, MR, and SO groups at 18-month-old). ^a^*p* ≤ 0.05 compared to aged control rats (24- month-old).

The impact of SO and MR hydrosols on anxiety-like behavior in both young and aged rats was investigated using the open field and dark/light box tasks, each conducted over a 10-min period. Aged rats exhibited a significant reduction in entries into the inner zone of the open field and fewer transitions between dark/light boxes in comparison to their younger counterparts (*p* < 0.0001) (Figures 5A,B). Notably, treatment with both SO and MR hydrosols resulted in a significant alleviation of these anxiety-like behavior (*p* ≤ 0.05).

**Figure 6:**
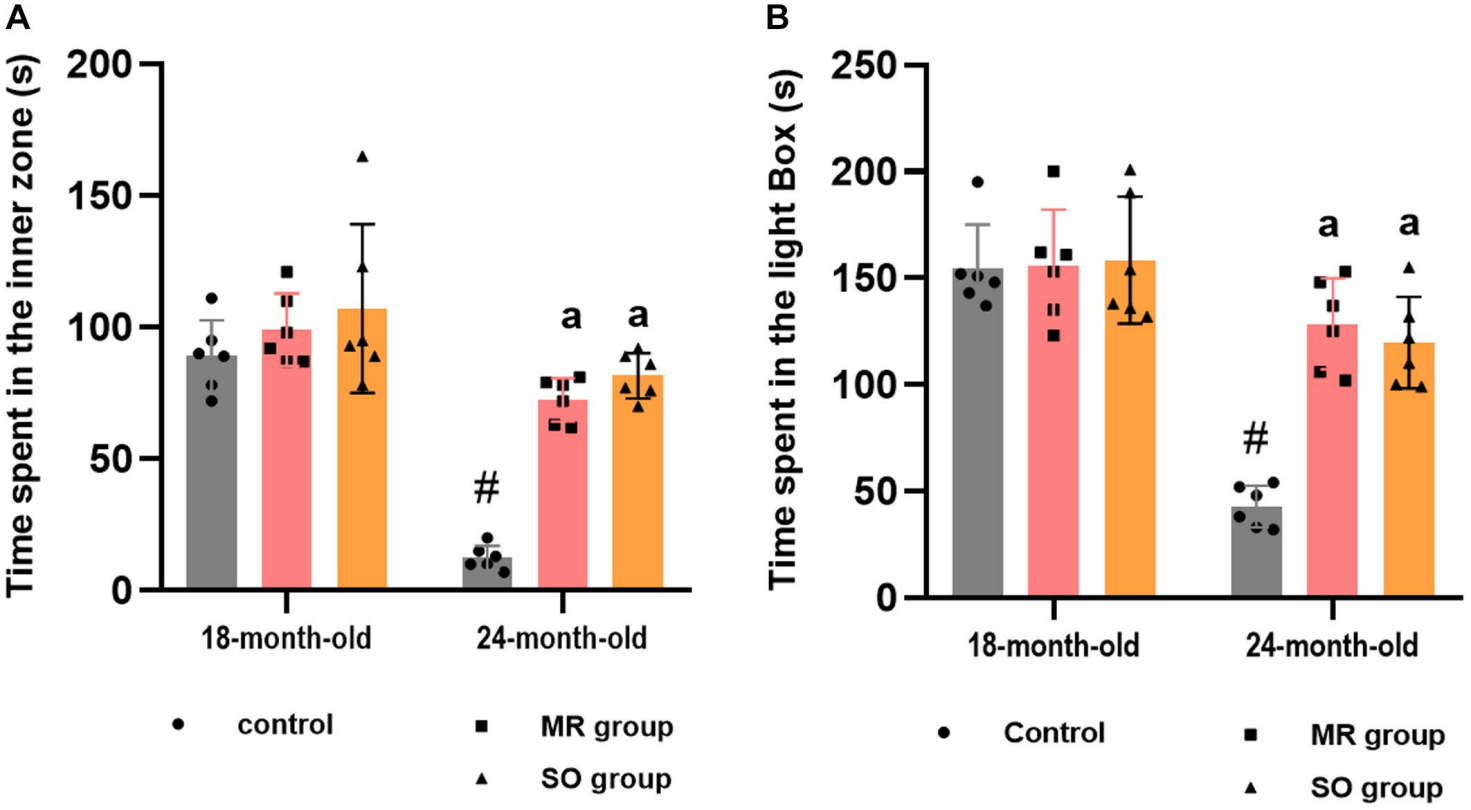
Effects of the hydrosols of *Salvia officinalis* (SO) and *Mentha rotundifolia* (MR) on the time spent in the inner zone by aging rats in the open field **(A)** and the time spent in the light box in the light/dark box task **(B)**. Results are presented as means ± SD (*n* = 6). ^#^*p* ≤ 0.05 compared to the young rat groups (control, MR, and SO groups at 18-month-old). ^a^*p* ≤ 0.05 compared to aged control rats (24- month-old).

Similarly, significant disparities were observed in the time spent within both the inner zone and the light box among the experimental groups (Figures 6A,B). Aged rats demonstrated a significantly reduced duration in both areas compared to their younger counterparts (*p* ≤ 0.0001), indicative of heightened anxiety levels. In contrary, the administration of SO and MR hydrosols to aged rats resulted in a substantial increase in the time spent in these areas (*p* < 0.0001 for both) suggesting a potent anxiolytic effect of the treatments.

#### 3.5.3 Hydrosols improve alternance and short-term spatial memory in aged rats

**Figure 7:**
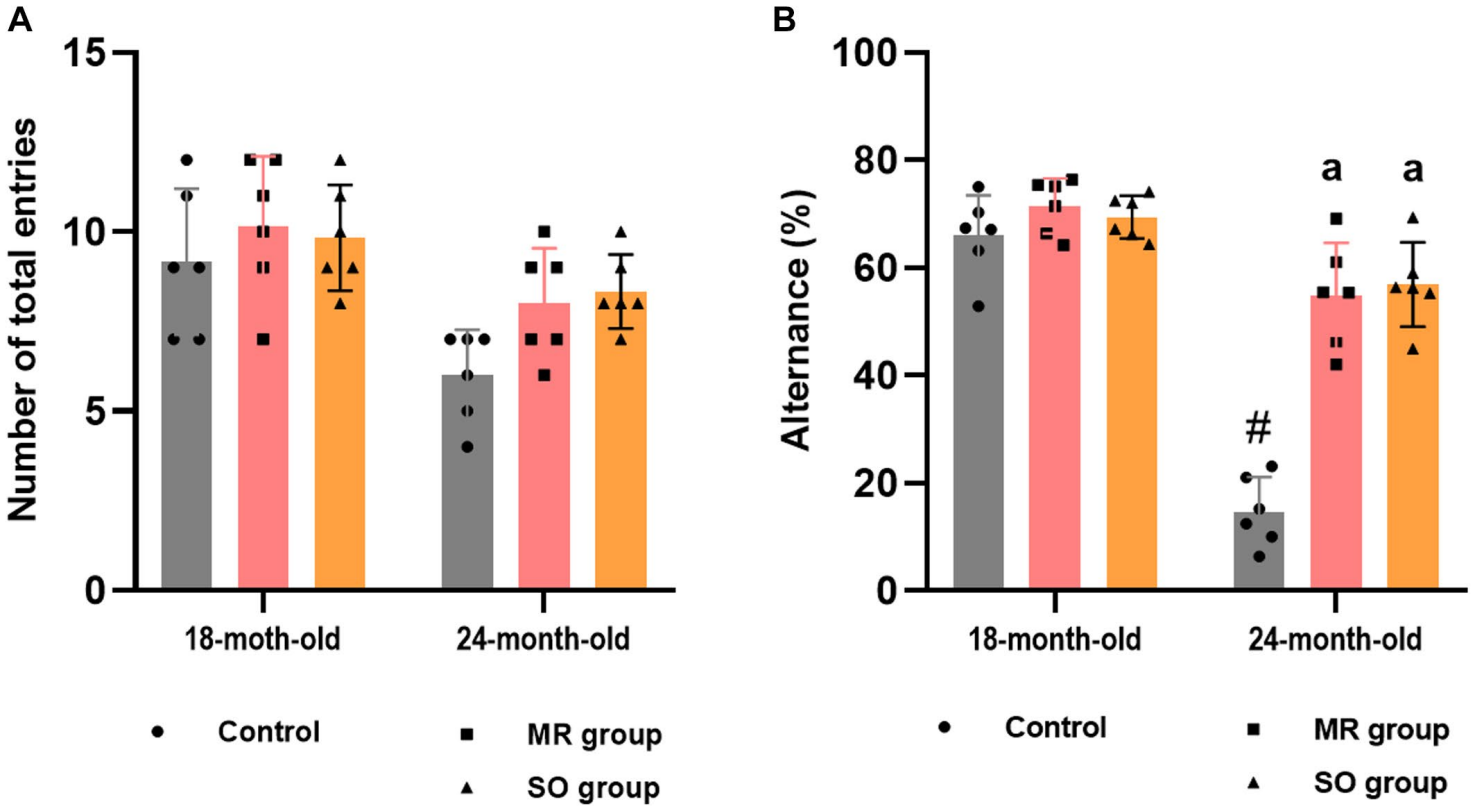
Effects of the hydrosols of *Salvia officinalis* (SO) and *Mentha rotundifolia* (MR) on aging-related deficiency on the spontaneous alternation y-maze in rats, presented as the number of total entries in different arms **(A)**, and percent alternations **(B)**. Results are presented as means ± SD (*n* = 6). ^#^*p* ≤ 0.05 compared to the young rat groups (control, MR, and SO groups at 18-month-old). ^a^*p* ≤ 0.05 compared to aged control rats (24- month-old).

**Figure 8:**
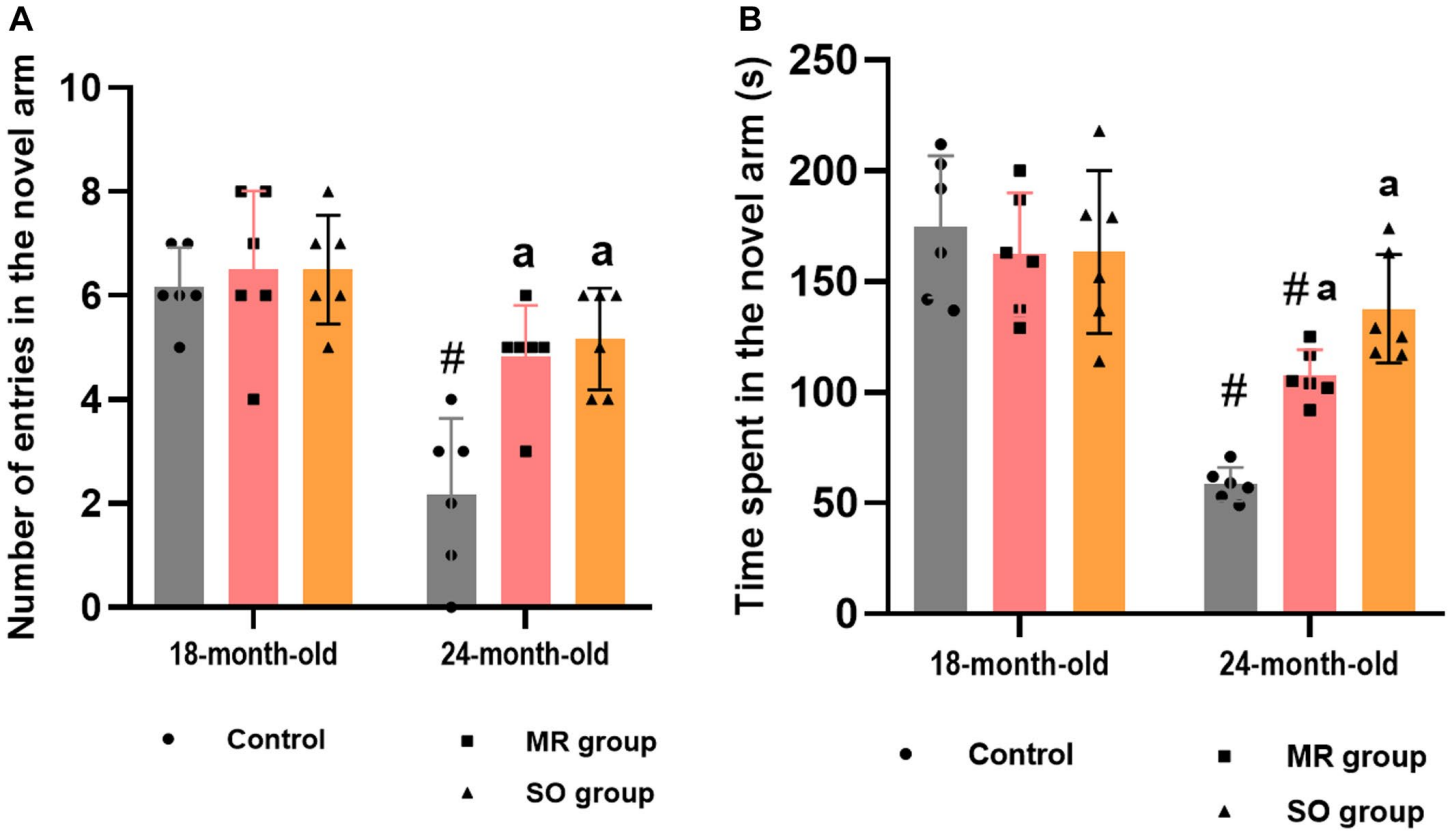
Effects of the hydrosols of *Salvia officinalis* (SO) and *Mentha rotundifolia* (MR) on the reference working memory of aging rats: the number of entries **(A)** and the time spent **(B)** in the novel arm in the y-maze task. Results are presented as means ± SD (*n* = 6). ^#^*p* ≤ 0.05 compared to the young rat groups (control, MR, and SO group at 18-month-old). ^a^*p* ≤ 0.05 compared to aged control rats (24- month-old).

The effect of MR and SO hydrosols on age-related exploratory activity and cognitive impairments was examined using the y-maze task. Assessment included the evaluation of spontaneous alternation behavior (Figure 7) and reference working memory, represented by the number of entries into the novel arm and the corresponding time spent in it (Figure 8). The results revealed a significant decrease in alternation in control aged rats that was reversed in MR and SO hydrosols treated groups (*p* < 0.0001) (Figure 7B), demonstrating a higher exploratory activity.

Regarding reference working memory, the outcomes indicated a significant reduction in both the number of entries into the novel arm and the corresponding time spent within it among aged rats (*p* < 0.0001), in contrast to their younger counterparts (Figures 8A,B). Treatment of aged rats with MR and SO hydrosols yielded a noteworthy enhancement in spatial working memory. This improvement was evident through a substantial increase in the number of entries into the novel arms when compared to the aged control group (*p* = 0.006 and *p* = 0.0015, respectively) and a heightened duration spent within the novel arm (*p* = 0.0361 and *p* = 0.0001, respectively).

### 3.6 Hyperalgesia and cold allodynia

**Figure 9:**
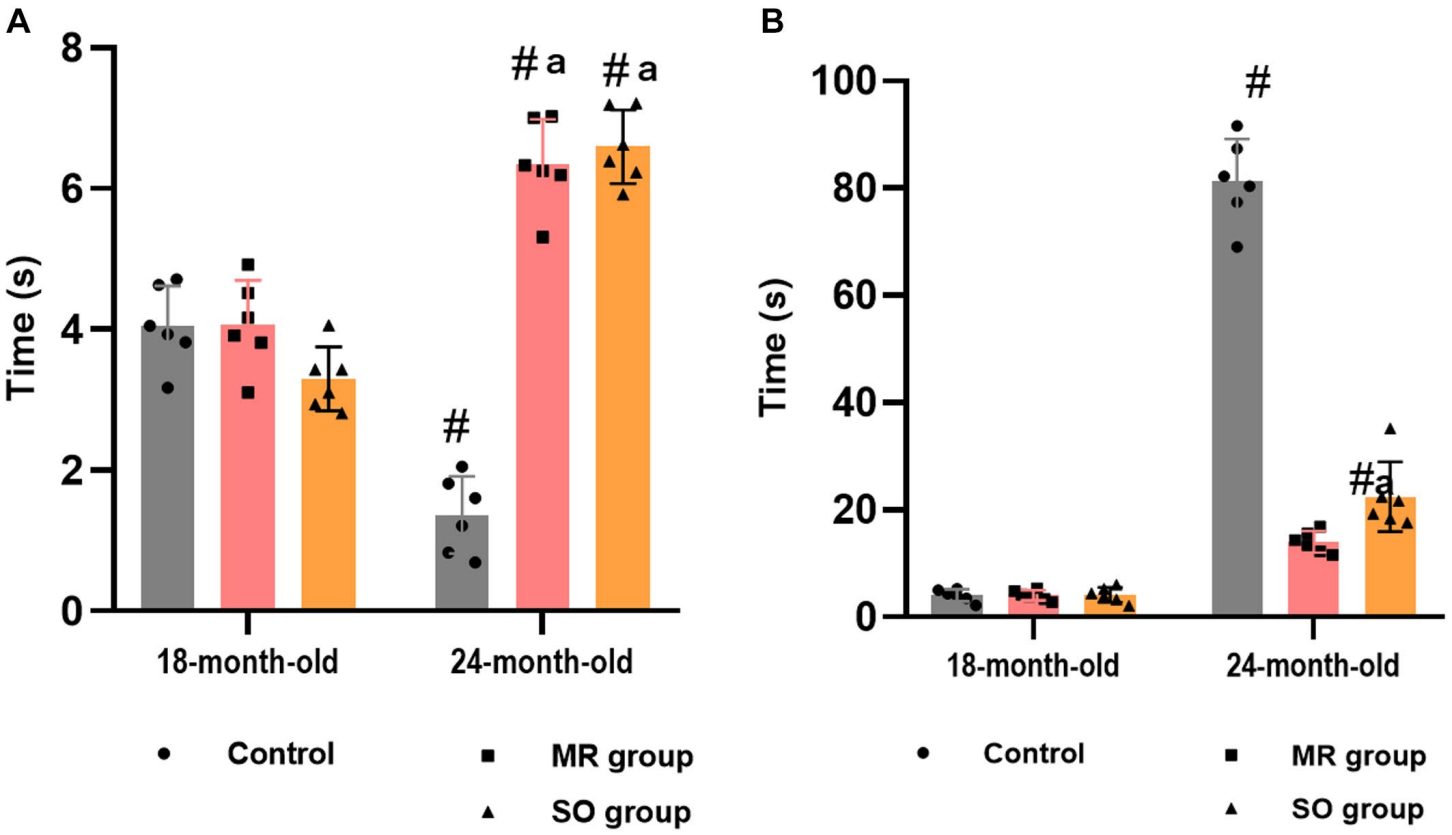
Effects of the hydrosols of *Salvia officinalis* (SO) and *Mentha rotundifolia* (MR) against hyperalgesia **(A)** and cold allodynia **(B)**. ^#^*p* ≤ 0.05 compared to the young rat groups (control, MR, and SO groups at 18-month-old). ^a^*p* ≤ 0.05 compared to aged control rats (24- month-old).

Pain sensitivity was evaluated through the tail immersion test. A notable age-related increase in animals’ pain sensitivity was evidenced by a reduction in tail withdrawal latency time within the control group (*p* ≤ 0.05) (Figure 9A). Groups treated with MR and SO hydrosols exhibited a substantial increase in tolerance to heat stimuli, as reflected by a significantly prolonged tail withdrawal latency time (*p* ≤ 0.05) (Figure 9A). These findings imply that MR and SO hydrosols exert an analgesic effect, suggesting their potential utility in mitigating age-associated changes in pain sensitivity.

Cold allodynia was assessed using the acetone test. Notably, a substantial augmentation in the duration of licking, biting, and paw lifting in response to the stimulus was observed in aged rats belonging to the control group (Figure 9B). In contrast, these behaviors exhibited a marked reduction in groups treated with hydrosols. Specifically, the behavior of the 24-month-old male rats treated with MR hydrosol closely resembled that of 18-month-old rats, indicating a potential ameliorative effect of the hydrosol on cold allodynia in aging rats (*p* = 0.0061) (Figure 9B).

### 3.7 Hepatic biomarkers concentrations

Aged rats within the control group exhibited significant elevated levels of AST, ALT, and GGT in comparison to groups treated with MR and SO hydrosols (*p* ≤ 0.05) (Table 4). However, no discernible difference was observed in the levels of bilirubin among the investigated groups (*p* ≥ 0.05).

**Table 4:** Effects of the hydrosols of *Salvia officinalis* (SO) and *Mentha rotundifolia* (MR) on hepatic biomarkers of aged rats.

Parameters	ALT	AST	GGT	Bilirubin
	UI/L plasma			
Control group	114.76 ± 13.15	86.35 ± 12.85	7.22 ± 2.34	1.72 ± 0.5
MR hydrosol	42.18 ± 2.81^a^	28.19 ± 3.61^a^	0.81 ± 0.36 ^a^	1.24 ± 0.18
SO hydrosol	38.63 ± 2.44^a^	26.79 ± 1.05^a^	3.07 ± 0.92^ab^	1.51 ± 0.36

Values are mean ± SD (*n* = 6). The results are considered significantly different for *p* ≤ 0.05. ^a^ in comparison with the standard, ^b^ in comparison with SO hydrosol.

### 3.8 Antioxidants levels in brain and liver tissues

The analysis of GSH, GPx, and SOD levels in brain and liver tissues revealed significant antioxidant effects of MR and SO hydrosols, Table 5. Both hydrosols groups exhibited elevated GSH levels, increased GPx activity, and enhanced SOD activity compared to the control group, indicating considerable defense against oxidative stress. Notably, the MR hydrosol treated group displayed particularly robust antioxidant responses in liver tissue. These findings suggest the potential of MR and SO hydrosols to confer antioxidant benefits.

**Table 5:** Effects of the hydrosols of *Salvia officinalis* (SO) and *Mentha rotundifolia* (MR) on antioxidants biomarkers in brain and liver tissues of aged rats.

Parameters	GSH (μmol/ g wet liver)	GPx (U/mg protein)	SOD (U/ mg protein)
Brain tissue			
Control group	1.32 ± 0.17	0.75 ± 0.08	29.94 ± 2.64
MR hydrosol	2.55 ± 0.4^a^	1.49 ± 0.08^a^	56.9 ± 3.34^a^
SO hydrosol	2.34 ± 0.48^a^	1.44 ± 0.1	57.16 ± 8.01^a^
Liver tissue			
Control group	4.87 ± 0.46	8.58 ± 0.7	45.45 ± 8.04
MR hydrosol	9.93 ± 0.78^a^	13.88 ± 0.51^a^	83.89 ± 11.87^a^
SO hydrosol	9.18 ± 0.5^ab^	12.97 ± 0.8^ab^	73.63 ± 7.97^a^

Values are mean ± SD (*n* = 6). The results are considered significantly different for *p* ≤ 0.05. ^a^ in comparison with the standard, ^b^ in comparison with SO hydrosol.

## 4 Discussion

Primarily composed of water and aromatic active principles, plant hydrosols are ideal candidates for chronic administration as soft functional drinks (Hamedi et al., 2017b). In this study, focus was directed toward *Mentha rotundifolia* (MR) and *Salvia officinalis* (SO) hydrosols that demonstrated noteworthy *in-vitro* antioxidant and anti-inflammatory activities, prompting us to investigate their potential as a long-term solution for preventing aging-related comorbidities.

The study design was conceived to match the human aging process. Laboratory rats, housed in cages with limited space and fed with processed food may replicate the sedentary behavior and imbalanced dietary patterns common in contemporary human lifestyles (Timlin et al., 2020). Such conditions are recognized for promoting oxidative stress, a key contributor to the aging process (Tan et al., 2018). Additionally, a day in the life of a rat approximates 34.8 days in the life of a human (Sengupta, 2013). Therefore, a six-month follow-up of 18-month-old animals is roughly analogous to a two-decade follow-up of a 50-year-old individual until the age of 70.

Aged rats displayed significant motor impairment assessed by the open field and rotarod tests. With advancing age, the loss of muscle mass, known as sarcopenia, can lead to weaker muscles, making movement and physical tasks more challenging (Volpi et al., 2004; Larsson et al., 2019). Numerous studies have linked oxidative stress to sarcopenia, demonstrating its role in increasing proteolysis and/or reducing protein synthesis (Meng and Yu, 2010; Wiedmer et al., 2021). Aged rats also manifested elevated anxious behaviors evaluated through the open field and dark/light box tests. Elevated levels of oxidative stress and reduced telomere length have been proven to be associated with anxiety disorders, both of which characterize aging (Simon et al., 2006; Pousa et al., 2021). Finally, aged rats exhibited cognitive deficits related to spontaneous alternation and spatial working memory evaluated by the Y-maze test. Indeed, the capacity for learning gradually diminishes with aging (Klencklen et al., 2017). The decline in memory retention capacity has been shown to result from oxidative stress-induced delayed-type apoptosis observed in the hippocampal CA1 region (Kandlur et al., 2020). In contrast, all observed behavioral impairments were averted in MR and SO hydrosols treated groups. The chemical composition of these hydrosols revealed the presence of various compounds that likely contributed to the deceleration of the aging process. 1,8 Cineole, documented for its robust antioxidant, anti-inflammatory, and anxiolytic properties, was found to exhibit anti-aging effect by inducing G0/G1 cell cycle arrest in stress-induced senescence in HepG2 cells (Juergens et al., 2004; Greiner et al., 2013; Dougnon and Ito, 2020; Rodenak-Kladniew et al., 2020). Camphor and camphene, along with 1,8 cineole, have been investigated for their anti-acetylcholinesterase and cognition enhancement effects (Werner et al., 2009; Babazadeh et al., 2023; Ertosun et al., 2023). Additionally, ɣ-terpinene and terpinen-4-ol have demonstrated neuroprotective effects against oxidative stress by inhibiting the generation of pro-inflammatory cytokines (Ayaz et al., 2017; Asikin et al., 2022; Deen et al., 2023).

As anticipated, aged rats demonstrated elevated pain sensitivity, as evaluated through the tail immersion test and exposure to acetone. The findings align with established knowledge indicating an age-related increase in pain threshold, predisposing individuals to neuropathic pain, characterized by hyperalgesia and allodynia (Yezierski, 2012; Lautenbacher et al., 2017). These effects were completely reversed in groups treated with MR and SO hydrosols showcasing a significant analgesic effect. The presence of camphor, a predominant component in both hydrosols, has been documented to alleviate hyperalgesia symptoms in various neuropathic pain models by modulating the excitability of dorsal root ganglion neurons (dos Santos et al., 2021; Li et al., 2023). Similarly, 1,8 cineole has been shown to inhibit the overexpression of the P2X2 receptor protein and mRNA, which plays a role in the transmission of algesia and nociception information by primary sensory neurons in the spinal cord and dorsal horn in CCI rats (Zheng et al., 2019).

Finally, the measurement of hepatic markers (ALT, AST, and GGT) as well as antioxidants (GSH, GPx, and SOD) in cerebral and hepatic tissues aligns consistently with the outcomes observed in behavioral tests. In accordance with the oxidative stress theory, the aged control rats manifested an augmented state of oxidative stress, as evidenced by a decline in antioxidant concentrations and a substantial elevation in ALT, AST, and GGT levels (Le Couteur et al., 2010; Kozakiewicz et al., 2019; Warraich et al., 2020). In contrast, administration of hydrosols effectively mitigated these adverse effects. The beneficial impact of hydrosols can be attributed to their role as a persistent source of antioxidants, potentially alleviating the age-related decline in liver detoxification functions. The intricate process of detoxification involves synergistic interactions between biotransformation enzymes and endogenous antioxidants. With progressive aging, a discernible reduction in endogenous antioxidant levels ensues, leading to a deceleration of the detoxification process (Vyskočilová et al., 2013). Consequently, the perturbation in the liver’s redox equilibrium and the ensuing oxidative stress culminate in the release of ROS and other deleterious molecules, including lipid peroxidation products and aldehydes, exerting systemic effects on distant organs and physiological systems (Adibhatla and Hatcher, 2010). Moreover, hepatic oxidative stress prompts the release of pro-inflammatory cytokines and mediators into the bloodstream (Dludla et al., 2020). These inflammatory signals possess the capacity to traverse the blood–brain barrier, contributing to neuroinflammation and influencing the antioxidant defenses within the cerebral milieu (Sankowski et al., 2015; Chen et al., 2022), as substantiated by our findings.

## 5 Conclusion

While aging remains an inexorable aspect of life, the primary objective of this study was not to impede its course but rather to attenuate the onset of consequential complications. The comprehensive evaluation encompassing physical, behavioral, and biochemical parameters revealed a compelling outcome. At the chronological age of 2 years, all animals exhibited discernible signs of aging. However, the groups treated with *Salvia officinalis* (SO) and *Mentha rotundifolia* (MR) hydrosols were biologically younger. These findings suggest that the hydrosols treatment holds promise in mitigating aging-related effects and offers a potential avenue for influencing the aging process. Our results contribute valuable insights for future research seeking interventions to promote healthier aging outcomes and address aging-related physiological changes.

## Data availability statement

The raw data supporting the conclusions of this article will be made available by the authors, without undue reservation.

## Ethics statement

The animal study was approved by Ethical Committee for Animal Veterinary Science and Public health, Institut Agronomique et Vétérinaire Hassan II, Rabat-Kingdom of Morocco. Approval No CESASPV_2023_A08. The study was conducted in accordance with the local legislation and institutional requirements.

## Author contributions

KB: Conceptualization, Data curation, Formal analysis, Funding acquisition, Investigation, Methodology, Project administration, Resources, Software, Supervision, Validation, Visualization, Writing – original draft, Writing – review & editing. HI: Data curation, Formal analysis, Investigation, Methodology, Writing – original draft. ZL: Conceptualization, Investigation, Methodology, Writing – original draft. MS: Investigation, Methodology, Software, Supervision, Validation, Visualization, Writing – original draft, Writing – review & editing. KT: Supervision, Validation, Visualization, Writing – review & editing.

## Footnotes

^1^ http://www.swissadme.ch/index.php
